# Continuous Finger Gesture Recognition Based on Flex Sensors

**DOI:** 10.3390/s19183986

**Published:** 2019-09-15

**Authors:** Wei-Chieh Chuang, Wen-Jyi Hwang, Tsung-Ming Tai, De-Rong Huang, Yun-Jie Jhang

**Affiliations:** 1 Department of Computer Science and Information Engineering, National Taiwan Normal University, Taipei 106, Taiwan; 60647012s@gapps.ntnu.edu.tw (W.-C.C.); 60447059s@ntnu.edu.tw (D.-R.H.); 60547051s@ntnu.edu.tw (Y.-J.J.); 2 NVIDIA Corp., 11001 Lakeline Blvd #100, Austin, TX 78717, USA; ntai@nvidia.com

**Keywords:** hand gesture recognition, wireless smart gloves, artificial intelligence, human machine interface, gated recurrent unit

## Abstract

The goal of this work is to present a novel continuous finger gesture recognition system based on flex sensors. The system is able to carry out accurate recognition of a sequence of gestures. Wireless smart gloves equipped with flex sensors were implemented for the collection of the training and testing sets. Given the sensory data acquired from the smart gloves, the gated recurrent unit (GRU) algorithm was then adopted for gesture spotting. During the training process for the GRU, the movements associated with different fingers and the transitions between two successive gestures were taken into consideration. On the basis of the gesture spotting results, the maximum a posteriori (MAP) estimation was carried out for the final gesture classification. Because of the effectiveness of the proposed spotting scheme, accurate gesture recognition was achieved even for complicated transitions between successive gestures. From the experimental results, it can be observed that the proposed system is an effective alternative for robust recognition of a sequence of finger gestures.

## 1. Introduction

Finger and hand gestures [1] posses rich information regarding human interaction and communication. The recognition of hand gestures is beneficial for intelligent human machine interfaces (HMI), where traditional input devices such as keyboards or mouses may not be required. In an intelligent HMI, finger gestures may be employed for the smart interaction for large varieties of applications. An example of the gesture-based HMI is the device control, where gestures can be viewed as commands for the operations of a device. Similarly, gestures can be regarded as signs for sign language translation. For virtual reality (VR) and augmented reality (AR) applications, gestures are adopted for the interaction between users and the digital environment. For these applications, accurate recognition of finger gestures is desired to implement the intelligent HMI.

A common solution to hand gesture recognition problems relies on cameras. The resulting techniques, termed vision-based gesture recognition (VGR) techniques, carry out gesture recognition on video sequences captured from cameras [2,3,4,5,6]. A common drawback of many VGR techniques is the high computation complexities for extracting gesture information from video sequences. For some HMI applications, such as smart device control, it is desired that the hand gesture recognition techniques are deployed in low-cost and low-power embedded or wearable devices with limited computation resources. Because of the high computation complexities, the implementation of realtime VGR-based recognition on embedded devices would be difficult.

Sensor-based gesture recognition (SGR) [7] techniques have been found to be effective alternatives to VGR techniques. Examples of sensors adopted for SGR techniques include electromyography, accelerometers, gyroscopes, flex, and/or photoplethysmography [8,9,10,11]. Some of these sensors can be deployed in embedded devices with low computation capacity for sensory data capturing and processing. With the growing popularity of wearable devices, SGR techniques are emerging as the major approaches for HMI.

Some existing SGR techniques [10,11] for hand gesture recognition have the shortcoming that only a single gesture can be recognized at a time. These techniques may not be directly applicable for continuous gesture recognition requiring the classification of a sequence of gestures. A challenging issue for continuous gesture recognition is gesture spotting, which aims to find the starting and end positions of each individual gesture. Accurate gesture spotting results are beneficial for isolating gestures so that each one can be recognized independently. In previous studies [8,12], user- or sensor-assisted gesture spotting operations have been adopted. Additional overheads could then be introduced.

A simple approach for automatic gesture spotting is based on the variances of sensory data. Samples with variances below a threshold are regarded as background [9]. The performance would then be dependent on the selection of thresholds. An alternative [13] is based on long short-term memory (LSTM) [14,15], which is a variant of a recurrent neural network (RNN) [15] capable of exploiting temporal dependency of input data. In addition to [13], the PairNet algorithm [16] has been found to be effective for gesture spotting. The PairNet algorithm is a special 1D convolution neural network (CNN) [15], where convolution layers with stride size 2 and kernel size 1×2 are adopted. As compared with the traditional 1D CNN approaches, the PairNet algorithm has the advantages of a wider receptive field and lower computational complexities for gesture spotting.

Although accurate spotting results have been observed in this framework [13,16], mobile phones equipped with accelerometers and gyroscopes are responsible for capturing sensory data. As a result, only the movements of hands holding mobile phones are spotted. However, in some applications, recognition of finger gestures may be more desirable. Finger gestures are usually characterized by diverse movements among different fingers, and complicated transitions between successive gestures. Therefore, the scheme in [13,16] for simple hand movements may not be well suited for the spotting of finger gestures.

The objective of the paper is to present a novel SGR system for the recognition of a sequence of finger gestures. The system is able to carry out accurate gesture spotting and recognition even for gestures with diverse movements and complicated transitions. The finger movements are captured by flex sensors [17,18], which measure the amount of deflection of each finger during the movements.

To collect and deliver the sensory data produced by the flex sensors, a wireless smart glove is implemented in the proposed SGR system. The glove consists of the flex sensors, an Arduino micro-controller, a battery module, and a wireless transmission module. The lithium polymer (LiPo) rechargeable battery [19] is used to supply power to the electronics components in the glove. The battery has the advantages of being light weight, having a high power density, and a large number of charge cycles. The LilyPad Arduino [19,20] is used as the micro-controller for data collection. It works on the LiPo battery, and allows easy connection with the other components in the glove. The wireless module supporting Bluetooth 4.0 is also included in the glove for the delivery of the collected data with a low power consumption [21]. Furthermore, e-textile techniques, such as conducting threads [22], are adopted for integrating/connecting these components.

In this study, a novel gesture spotting scheme based on the gated recurrent unit (GRU) [23,24] is proposed for the collection of sensory data produced by the smart glove. Similar to the LSTM technique, the GRU is a variant of RNN. While capable of exploiting temporal dependency of sensory data, the GRU has the additional advantages of lower computational complexities for inference operations. The training process for the GRU takes both the movements associated with different fingers and the transitions between two successive gestures into consideration. To facilitate the training operations, a novel labelling scheme is also proposed for the training data. In the scheme, each finger gesture and its associative transitions share the same label. In this way, the transitions can be included in the training process without introducing a high training overhead. Accurate gesture spotting can still be achieved with simple computation. On the basis of the spotting results, a maximum a posteriori (MAP) estimation is then performed for the final classification.

A prototype system based on the smart glove has been developed for performance evaluation. In the system, the training and testing operations are carried out on separate platforms. The server with GPU was adopted for the training process. The resulting GRU model was then implemented on a low-cost Raspberry Pi 3 platform for testing and evaluation. The experimental results reveal that the proposed algorithm is effective for hand gesture recognition at finger level which require both robust and accurate classification.

The remaining parts of this paper are organized as follows. Section 2 reviews some basic facts of the GRU for gesture spotting. The implementation of the smart glove for the finger gesture recognition is presented in Section 3. The proposed finger gesture recognition algorithm is given in Section 4. Transition issues are discussed in Section 5. The experimental results of the algorithm are included in Section 6. Finally, Section 7 contains some concluding remarks.

## 2. Preliminaries

**Figure 1:**
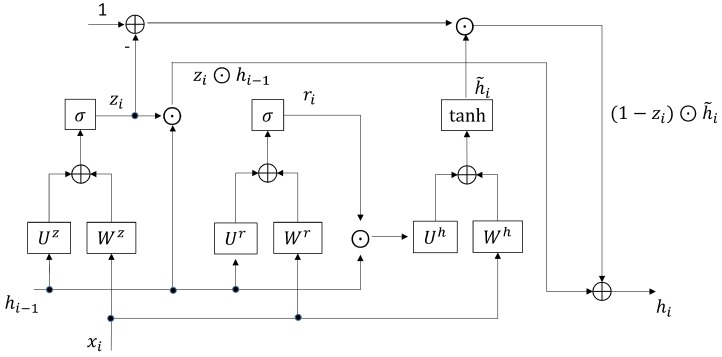
The summary of the operations in (1), (2), (3), and (4). These operations can be viewed as a function *F* given by (5), which takes $h_{i-1}$ and $x_{i}$ as the inputs, and produces output $h_{i}$. The bias vectors $b^{j},j=z,r,h,$ are omitted for the sake of simplicity.

This section provides a brief review of the GRU [23]. Consider an input sequence $X=\{x_{1},\ldots ,x_{T}\}$ to the GRU, where *T* is the length of the sequence. Let $H=\{h_{1},\ldots ,h_{T}\}$ be the state sequence associated with the GRU. All the states $h_{i},i=1,\ldots ,T,$ have the identical dimension *D*. With the initial condition $h_{0}=0$, *H* can be computed from *X* by

(1) $$\begin{array}{ccc} z_{i} & = & \sigma (W^{z}x_{i}+U^{z}h_{i-1}+b^{z}), \end{array}$$

(2) $$\begin{array}{ccc} r_{i} & = & \sigma (W^{r}x_{i}+U^{r}h_{i-1}+b^{u}), \end{array}$$

(3) $$\begin{array}{ccc} {\tilde{h}}_{i} & = & \tanh(W^{h}x_{i}+U^{h}\left(h_{i-1}⊙r_{i}\right)+b^{h}), \end{array}$$

(4) $$\begin{array}{ccc} h_{i} & = & \left(1-z_{i}\right)⊙{\tilde{h}}_{i}+z_{i}⊙h_{i-1}, \end{array}$$

for i=1,…,T, where $W^{j},$ and $U^{j},j=z,r,h,$ in (1), (2), and (3) are the weight matrices for input-to-hidden and hidden-to-hidden connections. We call $b^{j},j=z,r,h,$ the bias vectors. These matrices and vectors are the parameters to be learned during the training process. Furthermore, the function σ in (1) and (2) is an element-wise sigmoid function. The function tanh denotes the hyperbolic tangent. The operator ⊙ in (3) and (4) is the element-wise multiplication. Figure 1 summarizes the GRU operations in (1), (2), (3), and (4).

In the GRU, we call $z_{i}$ and $r_{i}$ the update gate and reset gate at the iteration *i*, respectively. The ${\tilde{h}}_{i}$ is the current memory content at iteration *i*. The update gate $z_{i}$ and reset gate $r_{i}$ determine the fraction of input information to be remembered and the fraction of the past information to be forgotten, respectively. We can see from (3) that the current memory content ${\tilde{h}}_{i}$ is determined by the past state $h_{i-1}$, the reset gate $r_{i}$, and the current input $x_{i}$ at step *i*. The current state $h_{i}$ is then computed by the current memory content $h_{i-1}$, the past state $h_{i-1}$, and the update gate $z_{i}$.

The operations in (1), (2), (3), and (4) can be regarded as a function *F*, which takes $h_{i-1}$ and $x_{i}$ as the inputs, and produces output $h_{i}$. That is,

(5) $$h_{i}=F\left(h_{i-1},x_{i}\right).$$

**Figure 2:**
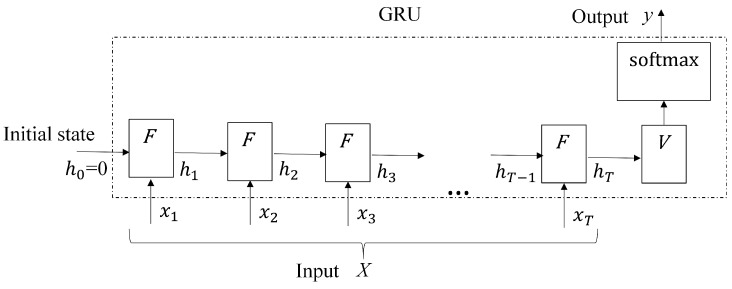
The complete gated recurrent unit (GRU) operations. There are *T* iterations for the input sequence *X*, where each iteration is represented by the function *F*. The output *y* of the GRU is then obtained from the result of the final iteration *T* by (6).

Given the input sequence *X*, the GRU then involves the iterative application of function *F* for each $x_{i}\in X$ from i=1 to i=T, as shown in Figure 2. At the current iteration *i*, the $x_{i}$ and $h_{i-1}$ are served as inputs, where the $h_{i-1}$ is the output produced at the previous iteration i−1. The output $h_{i}$ of the current iteration is then used as the input for the next iteration i+1. Let *y* be the result of GRU operations. In Figure 2, we see that

(6) $$y=\mathrm{softmax}(Vh_{T}),$$

where softmax denotes the softmax function, *V* is the state-to-output matrix, and $h_{T}$ is the output of function *F* at the final iteration *T*.

## 3. Sensory Data Acquisition

To capture the sensory data for the finger gesture recognition, a wireless smart glove equipped with flex sensors was implemented in this study.

### 3.1. Overview of the Wireless Smart Glove for Sensory Data Acquisition

**Figure 3:**
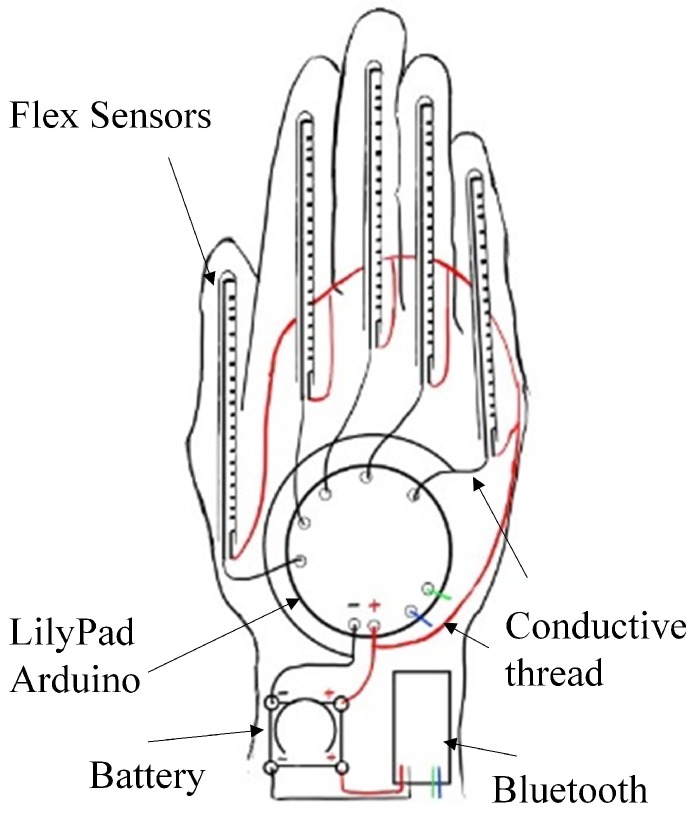
Layout of the wireless smart glove, which contains the flex sensors, Arduino Lilypad, Bluetooth module, conductive threads, and battery module.

**Figure 4:**
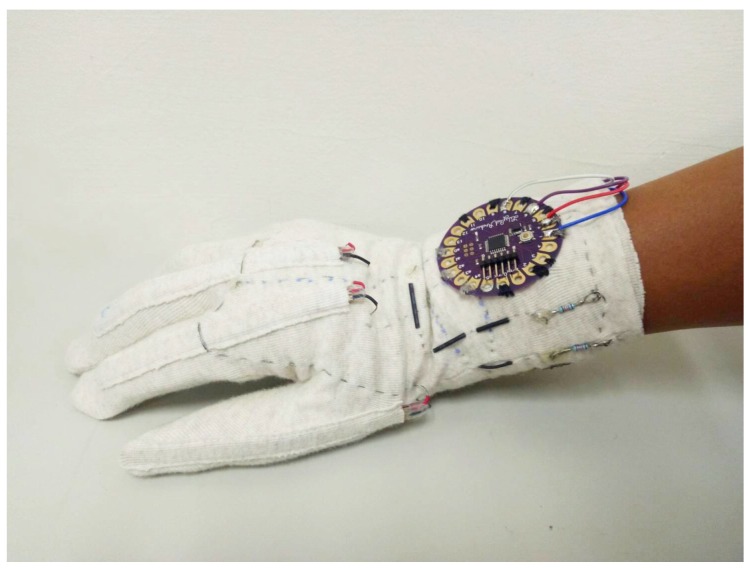
Side view of the wireless smart glove. No external power or wired data transmission are required.

Figure 3 shows the layout of the smart glove, which consists of flex sensors, Lilypad Arduino, Bluetooth module, conductive threads, and battery module. The specifications of the components are shown in Table 1. The side view of the glove is shown in Figure 4. No external power or wired data transmission are required by the glove. This could facilitate the deployment of the smart glove for sensory data acquisition. Note that the wireless smart glove is only responsible for data acquisition. The subsequent gesture recognition operations are carried out by other external devices receiving the data delivered by the glove.

### 3.2. Battery Module, Conductive Threads, Lilypad Arduino, and Bluetooth Module

The battery module contains a LiPo battery (Model No. LIR 2032) supplying power to the modules in the glove. The voltage provided by the battery is 3.6 V with capacity 70 mAh. The minimum cycle life is 500 cycles. Conductive threads with 3 ply are used to connect the modules. A dedicated flex sensor is assigned to each finger of the glove so that the movements of that finger can be recorded. The length of the flex sensors is 3.75 inches. The Lilypad Arduino acts as a micro-controller of the system. It is responsible for the collection of the data from the flex sensors. It operates at 16 MHz. A simple pre-processing operation is also carried out by the Lilypad Arduino for enhancing the robustness of the sensory data against interference. The collected sensory data is then delivered to external devices for gesture recognition by the Bluetooth module (Model No. HC-08) supporting Bluetooth 4.0 with low power consumption. The maximum baud rate is 9600 bps.

### 3.3. Flex Sensors

**Figure 5:**
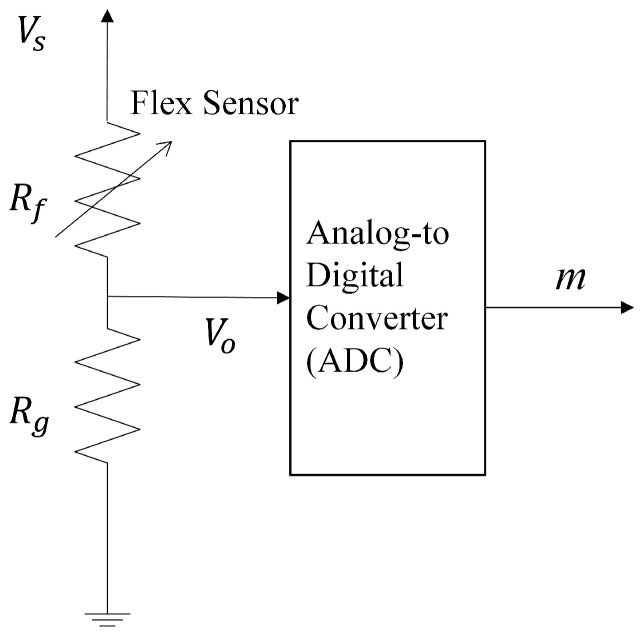
System for the acquisition of sensory data from a flex sensor, which can be viewed as a variable resistor with resistance value $R_{f}$. The other resistor has a fixed resistance value $R_{g}$. The $V_{s}$ and $V_{o}$ are the voltage supplied by the battery module, and the potential difference across $R_{g}$, respectively. The value *m* is the output of ADC when $V_{o}$ is its input.

The flex sensors can be viewed as variable resistors whose values are dependent on the degree of deflection of the sensors [18]. The range of the resistance values of the flex sensors is between 7 to 26 KΩ. A simple approach to acquire the sensory data produced by a flex sensor is shown in Figure 5, where $V_{o}$ is connected to the analog-to-digital converter (ADC) of the Lilypad Arduino. Let $R_{f}$ be the resistance value of the flex sensor. Therefore, 7 KΩ $\leq R_{f}\leq$ 26 KΩ. From Figure 5, the $R_{f}$ is related to $V_{o}$ by

(7) $$V_{o}=V_{s}\frac{R_{g}}{R_{f}+R_{g}},$$

where $V_{s}$ is the voltage provided by the battery module, and $R_{g}$ is the resistance value of the other resistor shown in Figure 5. It has a fixed value 13.3 KΩ (i.e., $R_{g}=$ 13.3 KΩ). The $V_{o}$ can be used as the sensory data. However, it is dependent on the source voltage $V_{s}$. Although the nominal voltage $V_{s}$ of the LiPo battery is 3.6 V, the voltage may vary from 4.2 V to 2.75 V, depending on the remaining capacity of the battery. Consequently, the variations in battery voltage may have impact on the sensory data. An alternative to $V_{o}$ is to find the resistance value $R_{f}$ of the flex sensor directly. Define

(8) $$B=\frac{V_{o}}{V_{s}}.$$

It can then be derived from (7) that

(9) $$R_{f}=\frac{\left(1-B\right)R_{g}}{B}.$$

The ratio *B* in (8) can be found by the ADC in Figure 5. Let *M* be the resolution of the converter (i.e., the number of output bits of the ADC), and let *m* be the output of ADC when $V_{o}$ is its input. The LilyPad Arduino provides ADCs with 10-bit resolution. Therefore, M=10. Because $V_{s}$ corresponds to the largest output value $2^{M}-1$ of the ADC, the ratio *B* can be approximated by

(10) $$B\approx \frac{m}{2^{M}-1}.$$

Because both *M* and $R_{g}$ are known a priori, the resistance value $R_{f}$ of the flex sensor can be computed from (9) by (10) when *m* (i.e., $V_{o}$) is available.

## 4. Gesture Spotting and Classification

After the data acquisition operations, the proposed system then processes the sensory data to produce the final classification results. The goal of this section is to provide a detailed discussions on the data processing aspects of the proposed system.

### 4.1. Overview of the Gesture Spotting and Classification System

**Figure 6:**
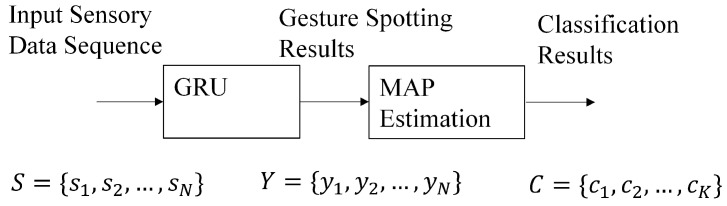
Overview of the proposed algorithm, where *S*, *Y*, and *R* are the input sensory data, gesture spotting results, and classification results, respectively.

On the basis of the sensory data produced by the wireless smart glove, we then carry out the gesture spotting and recognition operations, as shown in Figure 6. Given a sequence of sensory data $S=\{s_{1},\ldots ,s_{N}\}$ acquired from the smart glove, the gesture spotting operations produce spotting results $Y=\{y_{1},\ldots ,y_{N}\}$ using the GRU algorithm, where *N* is the length of the sensory sequence. Each sample $s_{t}\in S$ is the sensory data acquired at the time step *t*, t=1,…,N. All the samples in the sequence *S* are vectors. They have an identical dimension *L*, which is dependent on the sensors adopted for hand gesture recognition. Let *Q* be the number of gestures to be classified. Each sample $y_{t}\in Y$ is a vector with dimension *Q*. After *Y* is available, a post-processing operation based on MAP estimation is then carried out to obtain the final classification results *R*. Assume the sensory sequence *S* consists of data from *K* different gestures, where *K* is known a priori. The classification result *C* is then a sequence $C=\{c_{1},\ldots ,c_{K}\}$, where $c_{q}$, $1\leq c_{q}\leq Q$, is the index of the *q*-th gesture which appears in the sensory data sequence *S*. The platforms for gesture spotting and classification are outside the smart glove. As a result of the simplicity of the proposed algorithm, the platforms can be embedded systems with only limited computation capacity such as Raspberry Pi 3.

### 4.2. GRU-Based Gesture Spotting

**Figure 7:**
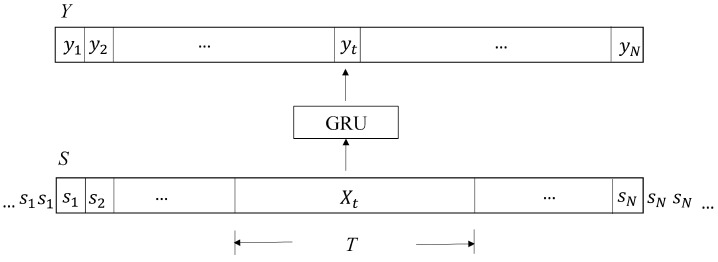
The gesture spotting operations based on the GRU. At the time step *t*, the $X_{t}$ is the input to the GRU, and $y_{t}$ is the result. The $X_{t}$ is a window of the sensory data *S*. It is centered at $s_{t}$ with length *T*.

The GRU algorithm operates on the sensory data sequence *S* in a sliding window fashion to obtain the gesture spotting results *Y*, as shown in Figure 7. Let $X_{t}$ be the window for the GRU operations producing $y_{t}\in Y$. The $X_{t}$ is centered at $s_{t}$ with length *T*, where T<N. When t<T/2 or t>N−T/2, parts of $X_{t}$ are outside *S*. These parts are filled with $s_{1}$ and $s_{N}$ for t<T/2 and t>N−T/2, respectively. To obtain $y_{t}$, we simply set *X* in Figure 2 as $X_{t}$. The resulting *y* in Figure 2 is then $y_{t}$. Starting from t=1, the operations stated above are repeated for each *t*, 1≤t≤N, for the computation of $y_{t}$ until t=N is reached. This completes the gesture spotting operations.

### 4.3. MAP-Based Post Operations

Let $y_{t,j}$, j=1,…,Q, be the *j*-th element of $y_{t}$. From (6), we see that $y_{t}$ is computed by the softmax activation function. The $y_{t,j}$ can then be viewed as the probability of the occurrence of the *j*-th gesture at time step *t*. Let $a_{t}$ be the index of the gesture having the largest probability at time step *t*. That is,

$$a_{t}=\underset{1\leq j\leq Q}{\mathrm{argmax}}y_{t,j}.$$

Define $A=\{a_{1},\ldots ,a_{N}\}$. We call *A* the path given the sensory data sequence *S*. In the proposed algorithm, we obtain the classification results $C=\{c_{1},\ldots ,c_{K}\}$ from the path *A* in accordance with the probability model given by

(11) $$P\left(C/A\right)=\prod_{q=1}^{K}P\left(c_{q}/A\right),$$

where

$$P\left(c_{q}/A\right)=\frac{|I_{c_{q}}|}{N},$$

and $I_{i}=\left\{a_{t}:a_{t}=i\right\}$. That is, $I_{i}$ is the set of time steps where the gesture *i* is the recognized gesture. The $|I_{i}|$ denotes the number of elements in $I_{i}$. That is, it indicates the number of occurrences of gesture *i*. The goal of MAP estimation is to find the classification result *C* maximizing P(C/A) in (11). This search process is equivalent to the identification of gestures which have top-*K* occurrence. The classification results $C=\{c_{1},\ldots ,c_{K}\}$ are then obtained from these gestures according to their locations in the path *A*.

## 5. Gestures and Transitions

This section addresses the labelling issues and proposed solutions for GRU training due to the transitions in a sequence of gestures. The gestures considered in this section only serves as examples to facilitate our discussion. The proposed labelling scheme can be directly applied to other finger gestures.

### 5.1. Finger Gestures

**Figure 8:**
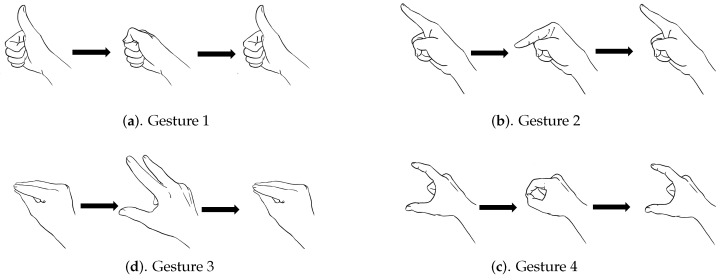
The four finger gesture classes considered in this study.

A finger gesture may have diverse movements among different fingers. To elaborate on this fact, Figure 8 shows the four finger gestures (i.e., Q=4) considered in this study. It can be observed from Figure 8 that, although both Gesture 1 and Gesture 2 involve only single-finger movements, they are based on different fingers. Furthermore, both Gesture 3 and Gesture 4 contain multiple-finger movements. In fact, they are characterized by different movements of thumb, index finger, and middle finger. To capture these diversified movements for effective GRU training, each finger is associated with a dedicated flex sensor for measuring the amount of deflection of that finger during the movements.

### 5.2. Transitions in a Sequence of Finger Gestures

**Figure 9:**
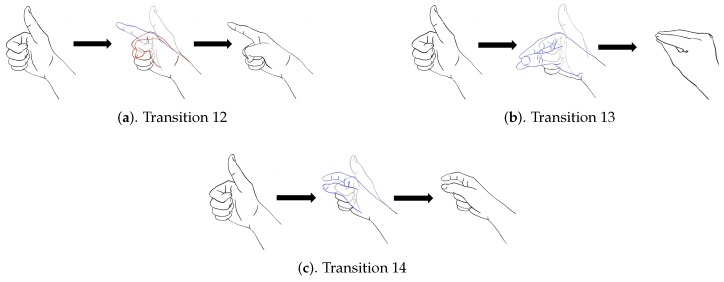
Three transitions associated with Gesture 1: Transition 12, Transition 13, and Transition 14, where Transition ij denotes transition from Gesture *i* to Gesture *j*.

**Figure 10:**
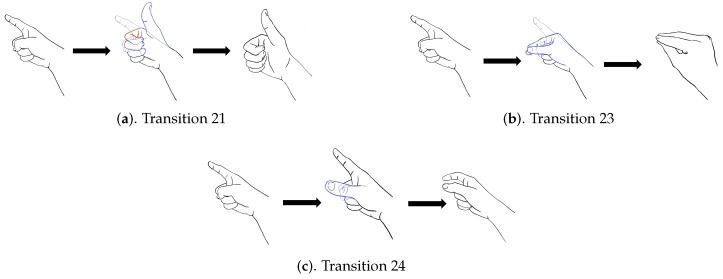
Three transitions associated with Gesture 2: Transition 21, Transition 23 and Transition 24, where Transition ij denotes transition from Gesture *i* to Gesture *j*.

**Figure 11:**
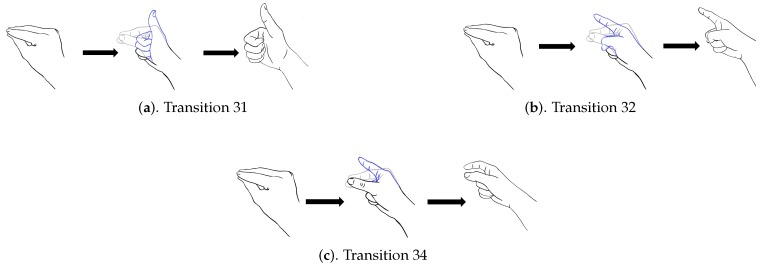
Three transitions associated with Gesture 3: Transition 31, Transition 32 and Transition 34, where Transition ij denotes transition from Gesture *i* to Gesture *j*.

**Figure 12:**
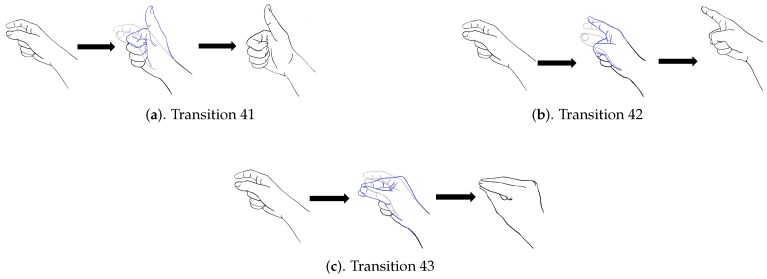
Three transitions associated with Gesture 4: Transition 41, Transition 42, and Transition 43, where Transition ij denotes transition from Gesture *i* to Gesture *j*.

In addition to diverse movements, the transitions are usually observed in a sequence of finger gestures, where the end position of fingers associated with a gesture may not be the same as the starting position of fingers associated with the subsequent one. The transitions provide additional movements for eliminating the discrepancy in finger positions between two successive gestures. Because there are four gestures in Figure 8, there are three transitions associated with each gesture. Figure 9, Figure 10, Figure 11 and Figure 12 reveal the transitions associated with Gestures 1, 2, 3, and 4, respectively. All these transitions can be also viewed as gestures. As shown in Figure 9, Figure 10, Figure 11 and Figure 12, the gesture marked Transition ij is the transition from Gesture *i* to Gesture *j*. There are Q(Q−1)=12 transitions.

### 5.3. Labelling Scheme for Finger Gestures

One simple approach for the recognition of a sequence of finger gestures using the proposed GRU technique is to ignore transitions and consider only gestures in Figure 8 for training. However, as shown in Figure 8, Figure 9, Figure 10, Figure 11 and Figure 12, the number of transitions is larger than the number of foreground gestures. By excluding the transitions from the GRU training, misclassifications of transitions as foreground gestures are likely. Another alternative is to treat each transition as a gesture with a distinctive label for training. In this way, transitions can be identified, and treated as background. Nevertheless, a large number of gestures are required to be classified in this approach. With Q=4 considered in Figure 8, the total number of gestures is then equal to Q+Q(Q−1)=16. The construction of complicated GRU networks would then be necessary.

In this study, we propose a novel approach for taking the transitions into consideration. In this approach, each Gesture *i* in Figure 8 and its associated transitions (i.e., Transition ij, j≠i) share the same label *i*. As shown in Table 2, the sensory data pertaining to Gesture *i* concatenated with Transition ij (denoted by Gesture *i* + Transition ij) have the same label as that of the sensory data pertaining to only the basic Gesture *i*. The transitions are therefore considered for training because they are labelled, and are included in the training sets. The GRU network still remains simple because gestures and transitions may share the same label. Both the effectiveness and simplicity of the proposed algorithm are advantageous for deploying the gesture recognition system on platforms with limited computation capacity for realtime inference.

## 6. Experimental Results

**Figure 13:**
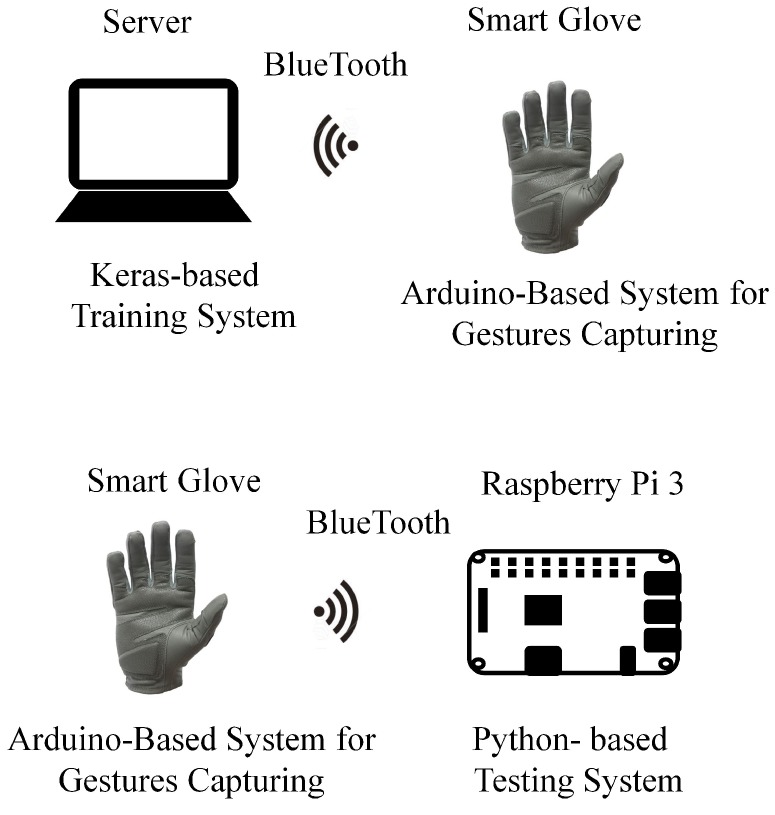
Setup of the experiments.

This section presents some experimental results of the proposed algorithm and system. Figure 13 shows the experimental setup in this study. The wireless smart glove shown in Figure 4 was used for the collection of sensory data for training or testing. A server with NVIDIA GTX 1070 GPU was adopted for the training of algorithms for the finger gesture recognition. The neural network models were built by Keras [25]. The inference model for testing was implemented by Python. The testing platform was different from the training one. It was based on a low-cost Raspberry Pi 3 computer. This could facilitate the deployment of the proposed systems for large varieties of internet-of-things (IOT) applications.

There were four gesture classes (Q=4) in the experiments, as shown in Figure 8. The transitions associated with class i,i=1,2,3,4, are shown in Figure 9, Figure 10, Figure 11 and Figure 12, respectively. The training set consisted of 2088 finger gestures from five participants. Some gestures in each class in the training set also contained transitions to the other classes. The gestures were labelled by the rules outlined in Table 2 for training. There were 2400 gestures from six participants in the testing set, which is different from the training set. Some gestures and their associated transitions in the test set formed a test sensory data. The number of gestures *K* in the test sensory data is known a priori. Table 3 shows the size of each gesture class of the training and testing sets.

Examples of applications of the proposed system with four gesture classes include the remote menu control of tablets or home appliances, and the authentication of IOT devices. Gestures acquired by the smart glove represent actions required by users or a personal identification number (PIN) to tablet or home appliances. The corresponding sensory data is delivered to Raspberry Pi 3 by wireless Bluetooth module, which then performs the continuous gesture recognition for subsequent actions. Because of its small size and low power consumption, the Raspberry Pi 3 can be easily configured as a tablet or a controller for home appliances. Table 4 shows the examples of gestures and their actions for various applications.

**Figure 14:**
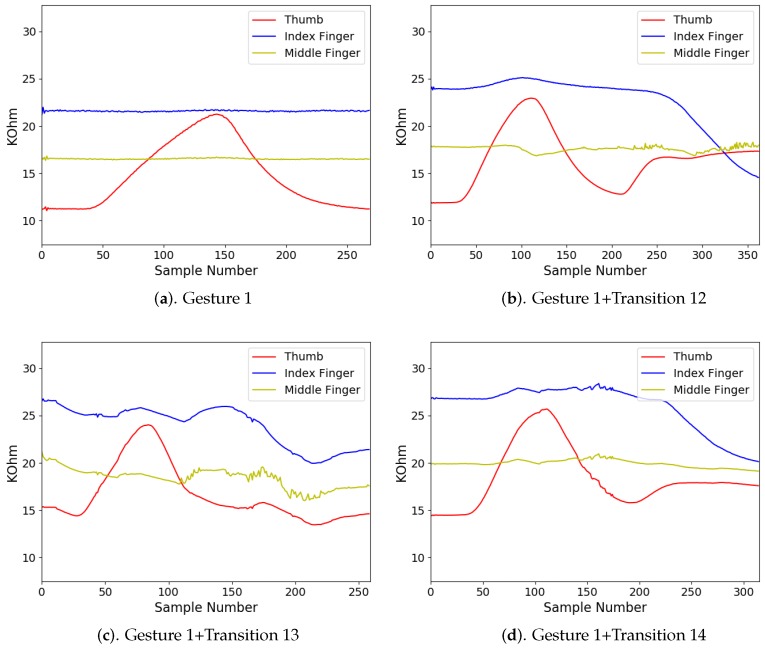
The measured sensory data produced by flex sensors of the smart glove for gesture class 1.

**Figure 15:**
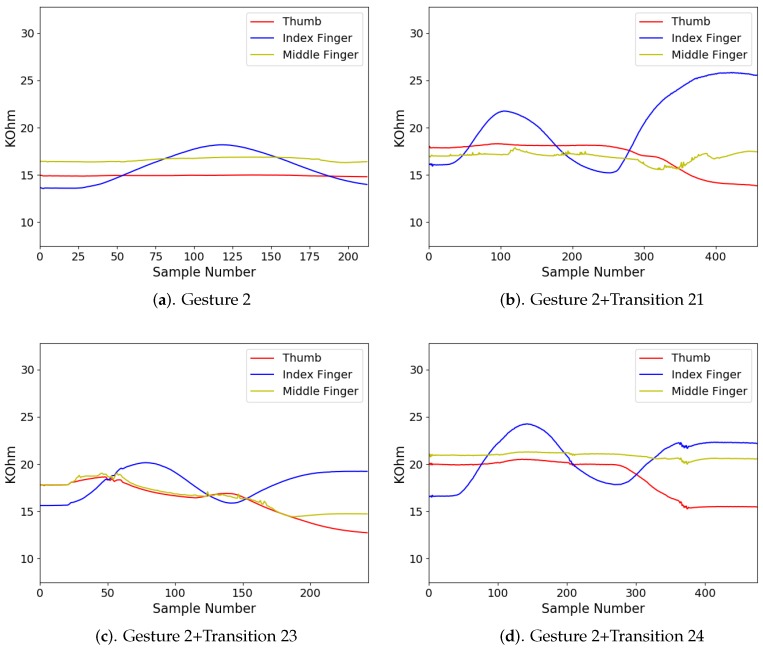
The measured sensory data produced by flex sensors of the smart glove for gesture class 2.

**Figure 16:**
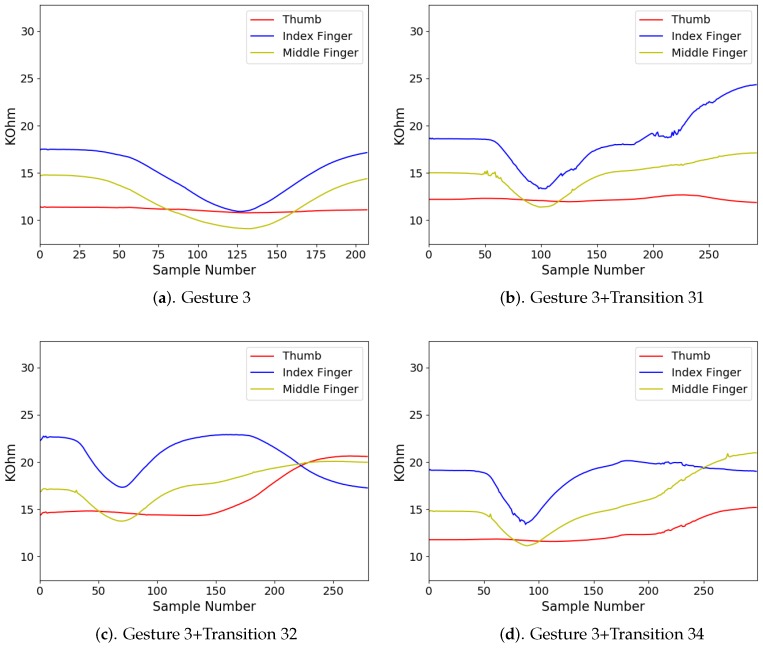
The measured sensory data produced by flex sensors of the smart glove for gesture class 3.

**Figure 17:**
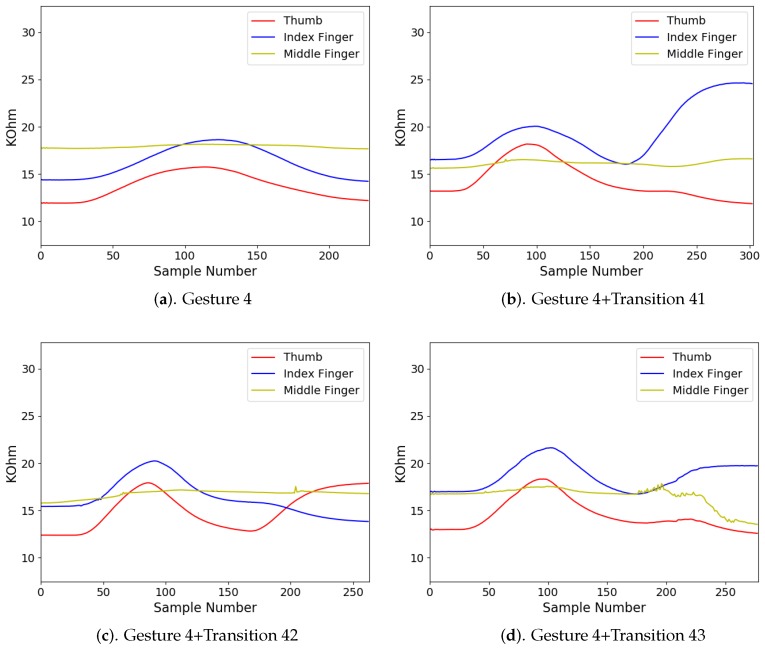
The measured sensory data produced by flex sensors of the smart glove for gesture class 4.

The measured sensory data produced by the flex sensors of the smart glove for each gesture class i,i=1,2,3,4, are shown in Figure 14, Figure 15, Figure 16 and Figure 17, respectively. For each class *i*, the sensory data for Gesture *i*, and Gesture *i* concatenated with Transition ij, j≠i, are revealed. Because the gestures in the four classes involve movements in thumb, index finger, and middle finger, the sensory data contains the resistance value $R_{f}$ of the flex sensors associated with these fingers in the smart glove. The resistance value of each sensor is sampled with sampling rate 50 samples/s.

It can be observed from Figure 14, Figure 15, Figure 16 and Figure 17 that the resistance value of each flex sensor is dependent on the finger movements of the corresponding finger. Therefore, gesture recognition based on flex sensors can be effective. Consider the sensory data shown in Figure 14 for Gesture 1 as an example. Only thumb movements are involved in Gesture 1. Therefore, without transition, we can see from Figure 14a that the waveform produced by the flex sensor associated with thumb exhibits large variations. Moreover, the large variations observed in the waveforms from other flex sensors in Figure 14b–d are mainly due to transitions. Therefore, it would be beneficial to include the transitions for training operations.

**Figure 18:**
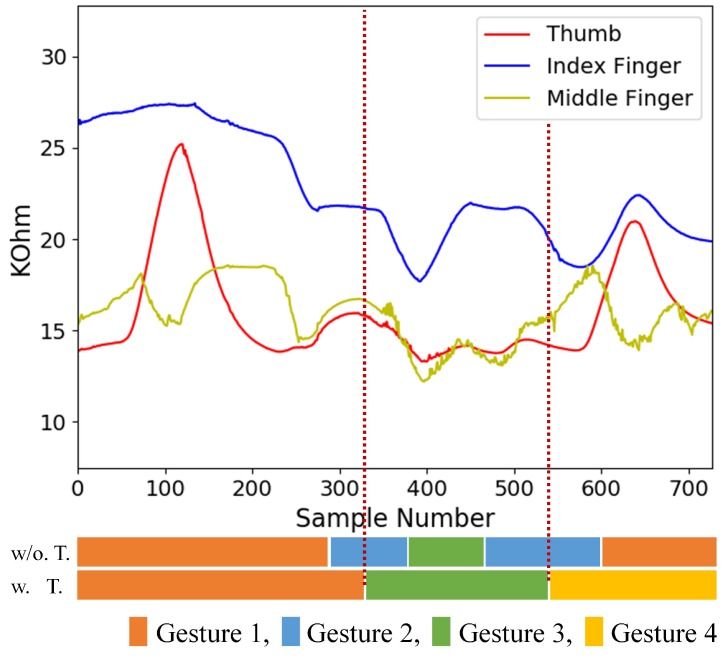
An example of a testing sequence produced by flex sensors consisting of three finger gestures (Gesture 1, Gesture 3, Gesture 4) back to back. The results of gesture spotting with and without the inclusion of transitions for training are shown at the bottom. The w. T. and w/o. T. denote the training operations with and without transitions, respectively.

**Figure 19:**
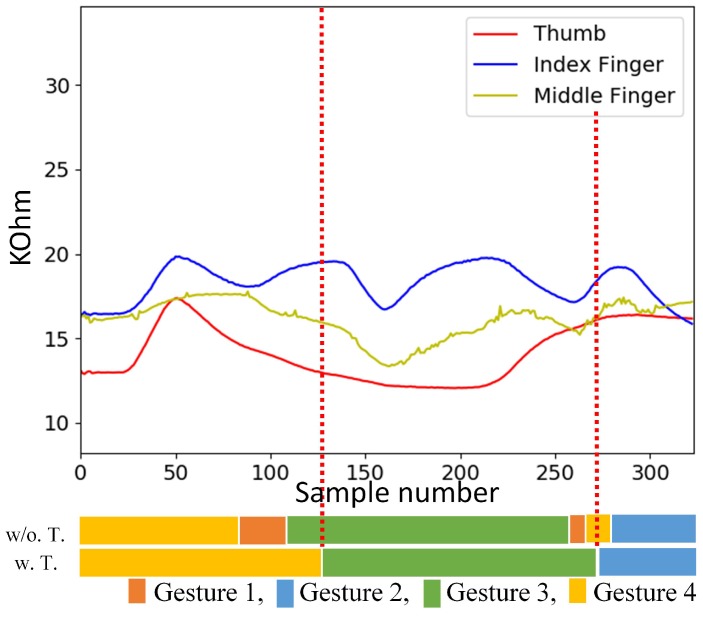
An example of a testing sequence produced by flex sensors consisting of three finger gestures (Gesture 4, Gesture 3, Gesture 2) back to back. The results of gesture spotting with and without the inclusion of transitions for training are shown in the bottom. The w. T. and w/o. T. denote the training operations with and without transitions, respectively.

Figure 18 and Figure 19 show examples of testing sequences produced by flex sensors consisting of three hand gestures back to back. The results of gesture spotting are also revealed as horizontal bars at the bottom of the figure. The bars labeled by w.T. and w/o. T. are the spotting results with and without inclusion of transitions for training, respectively. We can see from Figure 18 that, when the transitions are included for training, accurate gesture spotting can be achieved. The three gestures shown in Figure 18 are Gesture 1, Gesture 3, and Gesture 4. The algorithm identifies $I_{1}$, $I_{3}$, and $I_{4}$ as the largest sets. Therefore, the recognition outcome is $c_{1}$ = 1, $c_{2}$ = 3, and $c_{3}$ = 4. Furthermore, because the three gestures shown in Figure 19 are Gesture 4, Gesture 3, and Gesture 2, the corresponding recognition outcome is $c_{1}$ = 4, $c_{2}$ = 3, and $c_{3}$ = 2. By contrast, without considering the transitions, recognition outcomes are not correct due to the interferences by transitions.

The effectiveness of the proposed algorithm can be further demonstrated by evaluating the confusion matrix for the gesture recognition with and without inclusion of transitions for GRU training. Table 5 shows the evaluation results on the testing set. The size of the input window $X_{t}$ to the GRU is T=60. The dimension of the hidden state $h_{i}$ is D=128. The confusion matrix contains information about actual and predicted gesture classifications carried out by the system. Each element in a confusion matrix represents the percentage the gesture in the corresponding row is classified as the gesture in the corresponding column. Therefore, the diagonal elements in the matrix are the hit rates. The element in row *i* and column *i* is the hit rate of gesture *i*, denoted by $H_{i}$, which is the number of gestures in class *i* that are correctly classified divided by the total number of gestures in class *i*. From Table 5, we see that the proposed algorithm with the inclusion of transitions for GRU training has superior hit rates for all the four classes compared to its counterpart without the inclusion of transitions.

**Figure 20:**
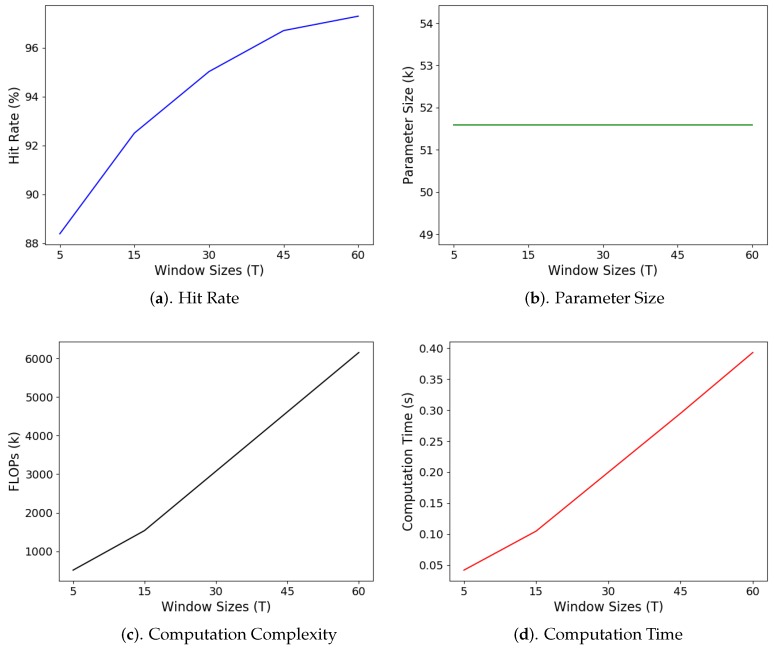
Average hit rate, parameter size, computation complexity, and average computation time of the GRU for different window sizes *T*. The average computation time is measured on the Raspberry Pi 3 platform. The dimension of the hidden states of the experiments was D=128.

The size of input window *T* may have an impact on the performance of the GRU. Figure 20 reveals the average classification hit rate, parameter size, computational complexity, and average computation time of the GRU for various input window size *T*. The average hit rate is defined as the number of gestures correctly classified divided by the total number of gestures in the testing set. The parameter size of the GRU is the total number of elements of matrices/vectors $W^{j},U^{j},b^{j},j=z,r,h,$ and *V* in (1), (2), (3), and (6). The computational complexity is measured as the number of floating point operations (FLOPs) for obtaining each output $y_{t}$. The average computation time is the average time required for carrying the inference of a single gesture in the testing set. It is measured on the Raspberry Pi 3 platform. In the experiments, the dimension of the hidden state was D=128. The training operations were carried out with the inclusion of transitions.

It can be observed from Figure 20a that GRUs with larger window sizes *T* have higher average hit rates than their counterparts with lower window sizes. This is because larger window sizes are beneficial for exploiting long-term dependency of the sensory data. On the other hand, a smaller window size is able to reduce computation time. This is because the computation complexity is lowered as the window size decreases, as shown in Figure 20c. Nevertheless, the average hit rate may be significantly degraded. In particular, when *T* is reduced from 60 to 5, we can see from Figure 20a that the average hit rate lowers from 97.30% to 88.38%. Furthermore, as revealed in Figure 20b, because the sizes of matrices/vectors in the GRU are independent of *T*, the GRUs with different window sizes have the same parameter size. Consequently, it may not be advantageous to decrease window size *T* to speed up the computation and/or reduce the parameter size.

**Figure 21:**
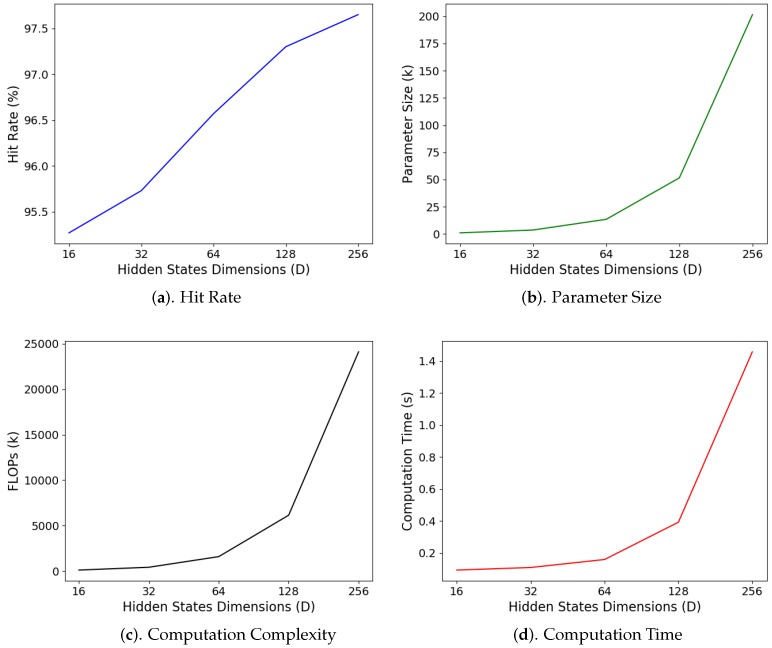
Average hit rate, parameter size, computation complexity, and average computation time of the GRU for different dimensions *D* of the hidden states. The average computation time is measured on the Raspberry Pi 3 platform. The window size of the experiments is T=60.

In addition to window size *T*, the dimension *D* of the hidden state $h_{i}$ is also influential on the performance of the GRU. The hidden state $h_{i}$ is responsible for abstracting the input sensory data for classification. Therefore, the selection of different dimensions may result in different average classification hit rates, parameter sizes, computational complexities, and computation times, as shown in Figure 21. In the experiments, the size of the input window *T* was 60. The training operations were carried out with the inclusion of transitions.

When D=256, the hidden states can accommodate more information for accurate classification. Therefore, it can be observed from Figure 21a that the proposed GRU algorithm with D=256 has the highest hit rate 96.65%. However, we can also see from Figure 21 that the parameter sizes, computation complexity, and average computation time grow with dimension *D*. This is because the sizes of matrices/vectors in the GRU are dependent on *D*. Therefore, when both the average hit rate and computation time are important concerns, we select the dimension to be D=128. In this case, this hit rate achieves 97.30% with a computation time of only 393 ms. By contrast, when D=256, the hit rate is 97.65% with a high computation time of 1457 ms. When it is desired to have the fastest computation time and smallest size for storing parameters, the dimension could be D=16 so that the computation time is only 94 ms at the expense of a slightly lower hit rate of 95.27%.

The comparisons of various algorithms for continuous hand gesture recognition are included in Table 6. It can be observed from the table that the proposed algorithm with the inclusion of transitions for training outperforms the other methods. In fact, its average hit rate compared to the testing set is 11.10% higher than that of its counterpart without the inclusion of transitions (i.e., 97.27% vs. 86.17%). Furthermore, it also has a superior hit rate compared to that of [13,16] by 9.82% (i.e., 97.27% vs. 87.45%) and 10.07% (i.e., 97.27% vs. 87.20%), respectively. The algorithms in [13,16] do not perform well because they aim at simple arm gestures without transitions. For the recognition of a sequence of finger gestures, the transitions usually occur between two successive gestures. Direct applications of the algorithms in [13,16] for finger gestures may then have an inferior performance to the proposed algorithm.

## 7. Conclusions and Future Work

We have built training and testing systems for the recognition of a sequence of finger gestures based on a wireless smart glove equipped with flex sensors. The testing systems are built on Raspberry Pi 3 computers so that the inference operations can be carried out on low-cost, embedded devices. Experimental results reveal that the wireless smart glove is able to effectively capture the finger movements. The GRU- and MAP-based techniques are able to provide accurate gesture spotting and classification. The novel labelling scheme for GRU-based gesture spotting operations is also beneficial for alleviating the interference introduced by transitions between successive gestures. In fact, the proposed GRU-based algorithm with the inclusion of transitions for training attains a hit rate of higher than 92% for the recognition of each class. Furthermore, it has an average hit rate 11.10% higher (i.e., 97.27% vs. 86.17%) than its counterpart without the inclusion of transitions on a testing data set consisting of 2400 gestures. The average computation time for the inference of a single gesture measure on the Raspberry Pi 3 platform is only 393 ms. The proposed system is therefore beneficial for HMI applications where reliable continuous hand gesture recognition on low-cost embedded systems for device control is desired.

A possible extension of the proposed work is gestures to text translation. For this application, a large number of gesture classes may be required. Furthermore, high classification accuracy may be necessary to convey correct text information. The requirement of high hit rates for the large number of gesture classes and/or long gesture sequences for the translation could be a challenging issue to be explored in the future.

## Figures and Tables

**Table 1:** Main Specifications of the components in the smart glove.

Components	Specifications
LilyPad Arduino	2.0–5.5 V Working Voltage
	ATmega328 Processor
	16 MHz Clock Rate, six 10-bit analog-to-digital converters (ADCs)
Flex Sensors	3.75 inch Active Length,
	Resistance Range 7 to 26 KΩ
Lipo Battery	LIR 2032, 3.2g Weight
	Coin Cell with 20 mm Diameter
	3.6 V Nominal Voltage, 70 mAh Capacity
	Minimum Cycle Life 500 Cycles
Bluetooth	HC-08, Bluetooth 4.0 Protocol
	9600 bps Maximum Baud Rate

**Table 2:** Labels associated with the sensory data considered in this study.

Label	Sensory Data
1	Gesture 1, Gesture 1 + Transition 12, Gesture 1+Transition 13, Gesture 1 + Transition 14
2	Gesture 2, Gesture 2 + Transition 21, Gesture 2 + Transition 23, Gesture 2 + Transition 24
3	Gesture 3, Gesture 3+ Transition 31, Gesture 3 + Transition 32, Gesture 3 + Transition 34
4	Gesture 4, Gesture 4 + Transition 41, Gesture 4 + Transition 42, Gesture 4 + Transition 43

**Table 3:** The size (in number of gestures) of each gesture class in the training and testing sets for the experiments considered in this study.

Gesture Class	Gesture 1	Gesture 2	Gesture 3	Gesture 4	Total
Training Set	504	559	531	494	2088
Testing Set	583	586	613	618	2400

**Table 4:** Examples of the gestures and their actions for various remote control applications. Each sequence for device control contains two gestures. Each sequence as a personal identification number (PIN) for internet-of-things (IOT) appliances authentication contains three gestures.

Applications	Gestures	Actions	Gestures	Actions
Tablets	Gesture 3+1	Menu Select	Gesture 4+1	Menu Scroll
	Gesture 3+2	Scale Up Screen	Gesture 4+2	Scale Down Screen
Music Player	Gestures 3+1	Volume Up	Gestures 4+1	Volume Down
	Gestures 3+2	Prev. Song	Gestures 4+2	Next Song
	Gestures 3+4	Power ON/OFF	Gestures 4+3	Play/Pause
TV	Gestures 3+1	Volume Up	Gestures 4+1	Volume Down
	Gestures 3+2	Prev. Channel	Gestures 4+2	Next Channel
	Gestures 3+4	Power ON/OFF	Gestures 4+3	Record ON/OFF
PIN	Gestures i+j+k	Authentication		

**Table 5:** Comparisons of the confusion matrix on the testing set for the proposed algorithm with (denoted by w. T.) and without (denoted by w/o. T.) the inclusion of transitions for training.

		Gest. 1	Gest. 2	Gest. 3	Gest. 4
Gest. 1	w/o. T.	94.75%	3.00%	1.80%	0.45%
	w. T.	99.70%	0.00%	0.00%	0.30%
Gest. 2	w/o. T.	0.70%	87.18%	1.83%	10.28%
	w. T.	0.85%	98.45%	0.42%	0.28%
Gest. 3	w/o. T.	5.61%	4.05%	89.30%	1.04%
	w. T.	0.52%	0.52%	98.43%	0.52%
Gest. 4	w/o. T.	2.81%	17.81%	5.33%	74.05%
	w. T.	0.84%	5.91%	1.26%	92.71%

**Table 6:** Comparisons of the hit rates of various algorithms on the testing data set for finger gesture recognition. The w. T. and w/o. T. denote the training operations with and without inclusion of transitions, respectively.

	$H_{1}$	$H_{2}$	$H_{3}$	$H_{4}$	Average
[13]	92.81%	91.84%	91.00%	74.75%	87.45%
[16]	95.92%	88.09%	90.80%	74.58%	87.20%
GRU w. T.	99.70%	98.45%	98.43%	92.71%	97.27%
GRU w/o. T.	94.75%	87.18%	89.30%	74.05%	86.17%

## Abbreviations

The following abbreviations are used in this manuscript:

ADC	Analog-to-Digital Converter
AR	Augmented Reality
CNN	Convolution Neural Network
FLOPs	FLOating Point operations
GRU	Gated Recurrent Unit
HMI	Human Machine Interface
IOT	Internet-Of-Things
LiPo	Lithium Polymer
LSTM	Long Short Term Memory
MAP	Maximum A Posteriori
PIN	Personal Identification Number
RNN	Recurrent Neural Network
SGR	Sensor-based Gesture Recognition
VGR	Vision-based Gesture Recognition
VR	Virtual Reality
w. T.	With Transition
w/o. T.	Without Transition
